# MR-Guided Focused Ultrasound Versus Radiofrequency Capsulotomy for Treatment-Refractory Obsessive-Compulsive Disorder: A Cost-Effectiveness Threshold Analysis

**DOI:** 10.3389/fnins.2019.00066

**Published:** 2019-02-07

**Authors:** Kevin K. Kumar, Mahendra T. Bhati, Vinod K. Ravikumar, Pejman Ghanouni, Sherman C. Stein, Casey H. Halpern

**Affiliations:** ^1^Department of Neurosurgery, Stanford University, Stanford, CA, United States; ^2^Department of Psychiatry and Behavioral Sciences, Stanford University, Stanford, CA, United States; ^3^Department of Radiology, Stanford University, Stanford, CA, United States; ^4^Department of Neurosurgery, University of Pennsylvania, Philadelphia, PA, United States

**Keywords:** obsessive-compulsive disorder, capsulotomy, neuroablation, radiofrequency, focused ultrasound, cost-effectiveness

## Abstract

Meta-analytic techniques support neuroablation as a promising therapy for treatment-refractory obsessive-compulsive disorder (OCD). This technique appears to offer a more favorable complication rate and higher utility than deep brain stimulation. Moreover, these pooled findings suggest that bilateral radiofrequency (RF) capsulotomy has marginally greater efficacy than stereotactic radiosurgery or cingulotomy. MR-guided focused ultrasound (MRgFUS) capsulotomy is an emerging approach with a potentially more favorable profile than RF ablation and radiosurgery, with preliminary data suggesting safety and efficacy. As a clinical trial is being developed, our study examined the cost and clinical parameters necessary for MRgFUS capsulotomy to be a more cost-effective alternative to RF capsulotomy. A decision analytical model of MRgFUS with RF capsulotomy for OCD was performed using outcome parameters of percent surgical improvement in Yale-Brown Obsessive Compulsive Scale (Y-BOCS) score, complications, and side effects. The analysis compared measured societal costs, derived from Medicare reimbursement rates, and effectiveness, based on published RF data. Effectiveness was defined as the degree to which MRgFUS lowered Y-BOCS score. Given that MRgFUS is a new therapy for OCD with scant published data, theoretical risks of MRgFUS capsulotomy were derived from published essential tremor outcomes. Sensitivity analysis yielded cost, effectiveness, and complication rates as critical MRgFUS parameters defining the cost-effectiveness threshold. Literature search identified eight publications (162 subjects). The average reduction of preoperative Y-BOCS score was 56.6% after RF capsulotomy with a 22.6% improvement in utility, a measure of quality of life. Complications occurred in 16.2% of RF cases. In 1.42% of cases, complications were considered acute-perioperative and incurred additional hospitalization cost. The adverse events, including neurological and neurobehavioral changes, in the other 14.8% of cases did not incur further costs, although they impacted utility. Rollback analysis of RF capsulotomy yielded an expected effectiveness of 0.212 quality-adjusted life years/year at an average cost of $24,099. Compared to RF capsulotomy, MRgFUS was more cost-effective under a range of possible cost and complication rates. While further study will be required, MRgFUS lacks many of the inherent risks associated with more invasive modalities and has potential as a safe and cost-effective treatment for OCD.

## Introduction

Obsessive-compulsive disorder (OCD) is a debilitating psychiatric disease that significantly impacts an individual’s social and occupational functioning. OCD has a lifetime incidence of 2–3% with onset typically occurring in late adolescence and early adulthood (Kessler et al., 2004). While cognitive behavioral therapy (CBT), exposure and response prevention (ERP), and pharmacological interventions are the mainstay of therapy for patients with OCD, approximately 40–60% of patients fail to respond to their first trial of therapy (Kreienberg et al., 2013). For those patients with severe treatment-refractory OCD, neurosurgical interventions such as neuroablation (ABL) or deep brain stimulation (DBS) remain therapeutic options (Bari et al., 2018).

ABL techniques conventionally consist of capsulotomy or cingulotomy using either radiofrequency (RF) ablation or stereotactic radiosurgery (SRS) (Brown et al., 2016). Alternatively, DBS for OCD alters the orbito-subcortical reward circuitry through electrical stimulation of targets, such as the anterior limb of the internal capsule, nucleus accumbens, ventral caudate, and/or ventral striatum (Abramowitz et al., 2009). Our group recently completed a meta-analysis of published data that revealed that ABL was more effective than DBS for OCD (Kumar et al., 2019). This analysis also identified non-significant trends indicating that capsulotomy was superior to cingulotomy and that RF ablation had greater efficacy than SRS. Our recent findings are of particular interest in the context of the emerging therapeutic modality for treatment of OCD: thermal lesioning using MR-guided focused ultrasound (MRgFUS).

MR-guided focused ultrasound is an attractive therapy compared to other neurosurgical interventions given its minimally invasive approach does not require craniotomy, sublesional test sonications can be delivered focally, and immediate lesioning can occur. Moreover, MRgFUS has been shown to be effective for other neurological disorders, such as essential tremor (Lipsman et al., 2013; Ravikumar et al., 2017). A recently published case series suggested MRgFUS was both safe and effective enough to compete with other surgical procedures for medication-resistant OCD (Jung et al., 2015). A meta-analysis examining MRgFUS for essential tremor revealed in-procedure complications consisting of dizziness (43.4%) and nausea or vomiting (26.85%) (Mohammed et al., 2018). Long term, ataxia (32.8%) and paresthesias (25.1%) were noted to be present at 3 months. Moreover, our group has previously projected the cost of MRgFUS in essential tremor (Ravikumar et al., 2017). However, it is unclear which combination of therapeutic results and associated procedure costs would permit MRgFUS to be a viable cost-effective alternative to other established neuroablative therapies, such as RF capsulotomy. RF capsulotomy was selected as the standard for comparison as it appears to have the highest utility among surgical therapies for OCD.

The present study used a decision-making analytical model to synthesize data from different sources in order to determine the cost-effectiveness of MRgFUS versus RF capsulotomy (Grutters et al., 2008). Given the limited clinical data evaluating outcomes of MRgFUS for OCD, we sought to determine the threshold at which MRgFUS yielded the same outcome as RF capsulotomy. This involved calculating the rate of success for MRgFUS that yielded the same utility as RF capsulotomy over a 1 year follow-up period.

## Materials and Methods

As mentioned above, we previously generated a decision-making analytical model to estimate and compare utility and complication rates of DBS and ABL for treatment-refractory OCD (Kumar et al., 2019). In this model, utility is a measure of relative patient preference for a given healthcare outcome (Weinstein et al., 1996). It is a parametric measure with values ranging between 0 (deceased) and 1 (perfect health). Each quality of life (QOL) score was converted to utility using published algorithms or linear extrapolation (Gold, 1996; Ravikumar et al., 2017). The quality-adjusted life year (QALY) combines utility with duration in a particular health state. This model projects the change in utility after surgery. Data for the model were derived from a critical review of published reports (Lipsman et al., 2013). The base case for this model was a patient with treatment-refractory OCD of average age and disease duration based on pooled data, who was considered a suitable subject for surgical intervention. Outcome parameters of interest were percent improvement in the Yale-Brown Obsessive Compulsive Scale (Y-BOCS) score from baseline, complications, and side effects of treatment (Goodman et al., 1989).

### Data Collection

We performed a PubMed search of articles reporting the results of RF capsulotomy for OCD. The search, performed in December 2016, included the term “obsessive-compulsive disorder” as a subject heading or title, combined with one of the following: “surgery” (subheading), “deep-brain stimulation (title or text), or any of the following in the title: lesion, RF, thermal, stereotaxic, stereotactic, or –otomy. Each article was reviewed by at least two authors. We excluded non-English language publications, case reports, reviews without original data, republication of previously reported data, animal studies, technical reports and other studies lacking original clinical data. The search was supplemented by employing the “Related citations” feature of PubMed and reviewing the bibliographies of review articles and selected publications. A recent review was consulted to help eliminate duplicated data (Brown et al., 2016).

Data extracted from each article included, where available, number of cases, study type, intracranial target, side(s) operated, number of operated cases, number of cases with complete follow-up, demographics, postoperative complications and side effects (type and number), mean follow-up duration, and pre- and post-operative mean Y-BOCS scores at the longest follow-up period provided. Complications were based on incidences only from reports documenting complications or those stating none occurred.

### QOL Correlations

Comparative effectiveness studies require utility or a similar metric of QOL. Using utility as a measure, change in Y-BOCS values were converted to mean improvement in QOL using a function based on published literature (Bystritsky et al., 1999; Volpato Cordioli et al., 2003; Sousa et al., 2006; Besiroglu et al., 2007; Norberg et al., 2008; Ross et al., 2008; Huppert et al., 2009; Hollander et al., 2010; Huff et al., 2010; Reddy et al., 2010; Simpson et al., 2010; Andersson et al., 2011; Srivastava et al., 2011; Farris et al., 2013; Ooms et al., 2014).

### Data Management

The mean response of Y-BOCS to RF capsulotomy was determined using random-effects and inverse variance-weighted meta-analysis of published observational data including mean age, sex distribution, duration of symptoms, and incidences of individual complications (Einarson, 1997; Nyman et al., 2001; Brown et al., 2016). Using meta-regression of studies measuring both changes in Y-BOCS and QOL, percent improvement in Y-BOCS was converted to mean improved utility (Bystritsky et al., 1999; Thompson and Higgins, 2002; Volpato Cordioli et al., 2003; Sousa et al., 2006; Besiroglu et al., 2007; Norberg et al., 2008; Ross et al., 2008; Huppert et al., 2009; Hollander et al., 2010; Huff et al., 2010; Reddy et al., 2010; Simpson et al., 2010; Andersson et al., 2011; Srivastava et al., 2011; Farris et al., 2013; Ooms et al., 2014; Kumar et al., 2019). In addition, a subtree of incidence of each reported complication and its effect on utility was constructed to determine the utility of the average patient with postoperative complications or surgical side effects from RF capsulotomy. This study adhered to the guidelines set forth by the Meta-analysis of Observational Studies in Epidemiology (MOOSE) group (Stroup et al., 2000). Beta distributions of probabilities and utilities were used to calculate model outputs.

### Analyses

The primary analyses sought to compare measured costs and effectiveness of bilateral RF capsulotomy with theoretical outcomes of MRgFUS. As little is known about MRgFUS effectiveness of durability, we utilized a threshold analysis over a 1-year follow-up period. Costs were derived from Medicare reimbursement rates for the procedures, a fair proxy for costs from the perspective of society. Sensitivity analysis of each MRgFUS parameter yielded cost, effectiveness (degree to which MRgFUS lowered Y-BOCS score and hence improved utility), and complication rate as the three most important parameters in determining the cost-effectiveness threshold between capsulotomy and MRgFUS. Parameters which were less effective included, incidence of individual complications, utility of individual complications, and time to onset of individual complications following MRgFUS. Accordingly, these were the parameters chosen to vary widely in a three-way sensitivity analysis. Statistical comparisons employed the Student’s *t*-test. Differences whose probabilities were less than 5% were considered significant. Meta-analytical pooling, meta-regression and statistical analysis involved Stata (v. 12⋅1, StataCorp, College Station, TX, United States). The decision analysis model employed TreeAge Pro 2017 (TreeAge Software, Williamstown, MA, United States).

## Results

### Data Collection

The literature search returned 533 abstracts. Based on the criteria outlined in the Section “Materials and Methods,” literature search identified eight publications, exclusive of individual case reports, containing complication and/or pre- and post-operative Y-BOCS scores (Nyman et al., 2001; Christensen et al., 2002; Oliver et al., 2003; Liu et al., 2008; Ruck et al., 2008; Csigo et al., 2010; D’Astous et al., 2013; Zhan et al., 2014). A total 162 subjects were identified in these studies, covering a period from 1999 to 2009.

### Cost and Effectiveness of RF Capsulotomy

The pooled average amount by which RF capsulotomy reduced preoperative Y-BOCS score was 56.6% and similar to a recent report (Rasmussen et al., 2018). This reduction in Y-BOCS score translates to a 22.6% improvement in utility. Operative complications of RF capsulotomy, along with the impact of each of these complications are detailed in Table 1. Complications occurred in 16.2% of RF cases. In 1.42% of these cases, complications, such as intracranial hemorrhage, were considered acute-perioperative and incurred additional hospitalization cost. The other 14.8% of cases, such as memory and cognitive decline, did not increase hospital reimbursement, although they affected utility. The costs of professional and facility reimbursements are summarized in Table 2. RF capsulotomy yielded a greater total reimbursement compared to MRgFUS. Subsequently, a rollback analysis of the RF capsulotomy tree yielded an expected effectiveness of 0.212 QALYs per year at an average cost of $24,099.

**Table 1:** Postoperative RF Complications—Pooled Frequency and Utility.

Category	%	*SD* (%)	Mean utility	*SD*	Utility reference
**Surgery-related**
Intracranial hemorrhage	1.42	1.00	0.75		Danish et al. (2005)
**Neurological**
Decreased memory	2.08	1.20	0.69		Neumann et al. (1999)
Cognitive decline	1.42	1.00	0.810	0.210	Klein et al. (2001)
Urinary incontinence	0.71	0.71	0.66	0.13	Castejon et al. (2015)
Abulia, apathy	4.26	1.70	0.6		Estimated
**Neurobehavioral**
Anxiety, related	4.96	1.83	0.604	0.017	Endicott et al. (2007)
Suicide	0.71	0.71	0	0	Sox et al. (2013)
Misc.	0.71	0.71	0.8		Estimated


*Incidence of operative complications of RF capsulotomy, along with the impact of each on utility and the relevant citation. OCD, obsessive-compulsive disorder; RF, radiofrequency*.

**Table 2:** Costs (2017 USD) of RF Capsulotomy and MRgFUS for OCD.

Treatment	CPT code	Professional reimbursement (USD)	DRG/APT/CPT (facility) code	Facility reimbursement (USD)	Total Reimbursement (USD)	
					Mean	*SD*
RF capsulotomy	61735, 76377	$1,721	76377	$32		
No major complications			24	$22,197	$23,950	$2,970
Major complications			23	$32,898	$34,651	$4,297
MRgFUS	0398T, 77290, 61800	$5,788	1537, 5611, 5612, 5613, 5614	$11,743	$17,660	$2,874


*Compilation of treatment modality, CPT code, professional reimbursement (USD), facility code, facility reimbursement (USD), and total reimbursement for RF capsulotomy and MRgFUS. OCD, obsessive-compulsive disorder; MRgFUS, magnetic resonance guided focused ultrasound; RF, radiofrequency*.

### Comparison of RF Capsulotomy to MRgFUS

**FIGURE 1:**
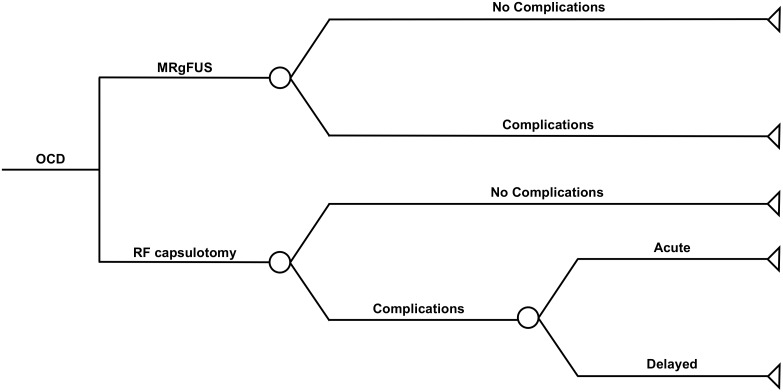
Decision tree comparing MgFUS and RF capsulotomy for treatment-refractory OCD. Possible outcomes of each treatment are listed. Acute complications of RF capsulotomy prolong hospital stays and increase costs. OCD, obsessive compulsive disorder; MRgFUS, magnetic resonance guided focused ultrasound; RF, radiofrequency.

Because no larger MRgFUS series for OCD have been reported other than Jung et al., sensitivity analysis was necessary to make a head-to-head comparison of the two treatments. This sensitivity analysis examined cost, utility, and complications in determine cost-effectiveness threshold for MRgFUS and RF capsulotomy. Using this approach, analysis revealed MRgFUS as the more cost-effective neurosurgical intervention for OCD under a wide range of possible outcomes (Figure 1).

## Discussion

**FIGURE 2:**
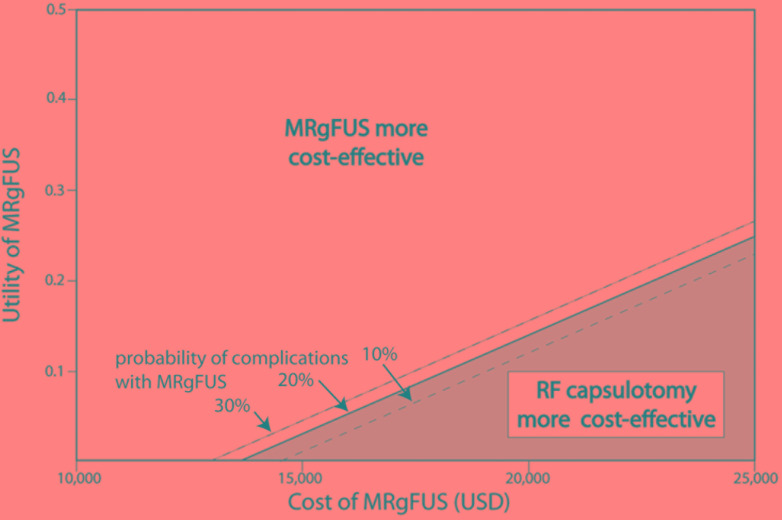
MRgFUS capsulotomy is a cost-effective therapy for treatment-refractory OCD under a wide range of parameters. Sensitivity analysis of each MRgFUS parameter yielded effectiveness (utility of MRgFUS), cost (USD) and complication rate (%) as the most important parameters in determining the cost-effectiveness threshold between MRgFUS and RF capsulotomy. OCD, obsessive-compulsive disorder; MRgFUS, magnetic resonance guided focused ultrasound; RF, radiofrequency.

Obsessive-compulsive disorder is a chronic and often disabling condition affecting millions of people, and neurosurgical interventions help many who do not benefit from other treatments. The psychiatrist-neurosurgeon Jean Talairach first described and performed the capsulotomy in 1949, and since then numerous technologies have emerged to safely perform this surgery (Zanello et al., 2017). MRgFUS is one such technology and potentially a more viable and cost-effective alternative to RF capsulotomy. Using a decision-making analytical model under multiple parameters of complication rate and procedure cost, these findings support the cost-effectiveness of MRgFUS over RF capsulotomy (Figure 2). These findings rely on the calculated utility of RF capsulotomy as determined by published data and reported complications. While the decision-making analytical model of MRgFUS versus RF capsulotomy provides an estimation of outcomes and potential utility of these procedures when limited data is available, they are not a replacement for randomized, controlled trials comparing these approaches directly.

Sensitivity analysis revealed three factors: cost, effectiveness, and complication rate as critical MRgFUS parameters determining cost-effectiveness, and there are multiple areas that could potentially enhance the cost-effectiveness of this procedure. As the MRgFUS complication rates and long-term impacts on utility remain to be examined, it is possible that effectiveness and costs of MRgFUS will change over time as this non-invasive technology improves and becomes widely available. Recent data from the essential tremor trial do reveal stable effects at 2 years post-operatively (Chang et al., 2017). MRgFUS is not expected to have the small but notable acute-perioperative complication rates seen with RF capsulotomy, which significantly increases hospitalization costs. Of note, some of the observed neurological and behavioral side effects with RF capsulotomy are secondary to lesion targeting. Thus, there exists the possibility that similar complications may be observed in MRgFUS for OCD. However, MRgFUS for essential tremor is known to have transient acute complications, such as headache, nausea and vestibular symptoms, the less invasive nature of the procedure may translate to a lower risk profile and contribute to increased cost-effectiveness, when compared to open procedures, such as RF capsulotomy (Jung et al., 2015).

MR-guided focused ultrasound is an innovative technology that possesses great potential as a safer and more cost-effective therapeutic intervention for treatment-refractory OCD. Future clinical trials for OCD should evaluate MRgFUS capsulotomy against other neurosurgical interventions, such as DBS, SRS, and RF ablation. These studies would benefit from careful assessment of the acute and long-term efficacy and complications of MRgFUS, the most important factors impacting the scalability of MRgFUS capsulotomy as a major therapy for OCD.

## Author Contributions

KK, MB, SS, and CH contributed to conception and design of the study. KK, SS, and CH organized the database and performed the statistical analysis. KK, MB, VR, PG, SS, and CH helped prepare and draft the manuscript.

## Conflict of Interest Statement

MB, PG, and CH have received research support from the Focused Ultrasound Foundation. The remaining authors declare that the research was conducted in the absence of any commercial or financial relationships that could be construed as a potential conflict of interest.
